# Modeling of Friction Phenomena of Ti-6Al-4V Sheets Based on Backward Elimination Regression and Multi-Layer Artificial Neural Networks

**DOI:** 10.3390/ma14102570

**Published:** 2021-05-15

**Authors:** Tomasz Trzepieciński, Marcin Szpunar, Ľuboš Kaščák

**Affiliations:** 1 Department of Materials Forming and Processing, Rzeszow University of Technology, al. Powst. Warszawy 8, 35-959 Rzeszów, Poland; 2 Doctoral School of Engineering and Technical Sciences at the Rzeszow University of Technology, Rzeszow University of Technology, al. Powst. Warszawy 12, 35-959 Rzeszów, Poland; d547@stud.prz.edu.pl; 3 Faculty of Mechanical Engineering, Institute of Technology and Material Engineering, Technical University of Košice, Mäsiarska 74, 040 01 Košice, Slovakia; lubos.kascak@tuke.sk

**Keywords:** coefficient of friction, friction, sheet metal forming, strip drawing test

## Abstract

This paper presents the application of multi-layer artificial neural networks (ANNs) and backward elimination regression for the prediction of values of the coefficient of friction (COF) of Ti-6Al-4V titanium alloy sheets. The results of the strip drawing test were used as data for the training networks. The strip drawing test was carried out under conditions of variable load and variable friction. Selected types of synthetic oils and environmentally friendly bio-degradable lubricants were used in the tests. ANN models were conducted for different network architectures and training methods: the quasi-Newton, Levenberg-Marquardt and back propagation. The values of root mean square (RMS) error and determination coefficient were adopted as evaluation criteria for ANNs. The minimum value of the RMS error for the training set (RMS = 0.0982) and the validation set (RMS = 0.1493) with the highest value of correlation coefficient (*R*^2^ = 0.91) was observed for a multi-layer network with eight neurons in the hidden layer trained using the quasi-Newton algorithm. As a result of the non-linear relationship between clamping and friction force, the value of the COF decreased with increasing load. The regression model F-value of 22.13 implies that the model with *R*^2^ = 0.6975 is significant. There is only a 0.01% chance that an F-value this large could occur due to noise.

## 1. Introduction

Sheet metal forming (SMF) is a process by which sheet metal parts are subjected to geometric change without material reduction. The process creates a force that causes the material to deform. Many physicochemical processes take place during forming which play a key role in the quality of the product obtained, the tooling lifetime or the accuracy of the shape of the formed product [1,2,3]. There are phenomena in the contact zone that are impacted by many variables, such as the macro- and microgeometry of the contact surfaces, pressure, processing temperature, type and viscosity of the lubrication used, die structure, topography and physicochemical phenomena of the contact surfaces, and load dynamics [4,5,6].

To determine the effects of friction in SMF, it is necessary to determine the coefficient of friction between the interacting elements [7,8]. In SMF, one cannot limit the description of the friction phenomenon to the Coulomb model, because there are phenomena such as adhesion and plowing [9,10]. The coefficient of friction is primarily determined by the roughness of the surface as well as the structure of the surface layer and its composition [11,12]. The value of the coefficient of friction (COF) is a variable value, and it depends on, among other factors, the pressure force applied [13]. In the case of the Grade 5 titanium alloy (Ti-6Al-4V) studied in this paper, the tribological properties of this material are not only affected by the processing method, but also by the distribution of the mixed crystal system α and β [14] and the content of the alloying elements aluminum and vanadium [15,16]. However, titanium and its alloys are characterized by poor tribological behavior in terms of a strong tendency to seize [17], severe adhesive wear [18,19], high and unstable friction, low resistance to abrasion [20,21] and susceptibility to fretting wear [22,23].

To protect the environment, the use of mineral oil-based lubricants should be limited due to their non-biodegradable nature and inherent toxicity. After forming, mineral oils must be eliminated before painting, which generates contaminated wastes through the use of volatile organic solvents [24]. Vegetable oil-based lubricants can be an ecological alternative to commonly used synthetic oils. Vegetable oils are non-toxic biodegradable substances, and due to the presence of long chains of fatty acids, they ensure good lubrication under boundary friction conditions [25,26]. They also show most of the properties required for lubricants destined for cold metal forming, such as good lubricity and a high viscosity index [27,28]. 

Multivariate regression analysis has also found many applications in the tribology of materials [29]. The significant parameters making a major contribution to the coefficient of friction of titanium carbide (TiC) reinforced metal matrix composites were identified using Analysis of variance (ANOVA) [30]. Evin et al. [31] modelled the COF of DC05 steel sheet using linear regression. However, the results obtained show that the analytical model appears to be more suitable than the regression model for the determination of the COF. Lüchinger [32] successfully determined an optimal friction model for bulk metal forming using stepwise nonlinear regression. Kumar and Kumaraswamidhas [33] have applied ANOVA to obtain the most significant parameter of AA 6061 composites subjected to the pin-on-disk tribological test. Wahyudi et al. [34] used ANOVA analysis at a standard significance level of *α* = 0.05 to analyse the COF of ultra-high molecular weight polyethylene determined in the pin-on-disc test. Trivedi and Bhatt [35] studied the tribological parameters of the cylinder liner/piston ring under sliding contact in the presence of lubricant. It was found that the wear character of the worn-out surface was significantly dependent on the load condition. Ambigai and Prabhu [36] conducted an ANOVA to study the significance of aluminum alloy composite parameters affecting the wear characteristics. The analysis allows one to find the relationship between normal load, sliding distance and the COF. Kalel et al. [37] used ANOVA to study the tribological behaviour of 17-4 PH stainless steel under different friction test conditions, i.e., duration of sliding, load and speed.

In recent years, in addition to the currently used static tests, methods of analysis that use artificial neural networks (ANNs) have gained great popularity [38,39]. They are successfully used to analyse technological processes and phenomena occurring within them. Eren et al. [40] used artificial neural networks to describe the friction stir welding process. Yan and Chen [41] used adaptive control for the optimization of the free forging process, where neural networks were used to modify proportional–integral–derivative controller settings and increase machining accuracy. Zhu et al. [42] used deep neural networks to describe the mechanical properties of steel depending on the alloy additives used and heat treatment parameters. The comparative study by Tyagi et al. [43] showed that the predictive ability of ANN is more efficient and fits in better with the experimental values than the response surface methodology model.

In recent years, an increasing number of tribological studies have turned to the use of ANNs [44,45]. Bhaumik et al. [46] used the ANN approach to find a biolubricant with optimised characteristics. Humulnicu et al. [47] analysed the use of ANNs to design lubricants with significantly lower COF. They considered the optimization of mixtures of diesel oil with rapeseed and sunflower oils for use in diesel engines. Boidi et al. [48] employed radial basis function neural networks to predict the COF in lubricated contacts with textured surfaces. It was shown that hardy multiquadratic radial basis functions provided satisfactory overall correlation with the experimental results. Trzepieciński and Lemu [49] applied genetic algorithms to optimize neural networks for the tribological strip drawing test. The results obtained have demonstrated that a genetic algorithm can successfully be applied to optimizing the training set to agree with experimental data. The use of ANNs in tribology has been discussed by Rosenkranz et al. [50] and Argatov [51].

The advantage of neural networks is the ability to predict the output signal based on the input data. This article presents the application of backward elimination regression and multi-layer ANNs for the prediction of the COF of Ti-6Al-4V titanium alloy sheets. As training data, use was made of the results of strip drawing tests conducted for different kinds of synthetic and biodegradable vegetable oil-based lubricants and variable load. It was found by experiment that as a result of the non-linear relationship between clamping and friction force, the value of the COF decreased with increasing load. The ANNs performance has been assessed based on different training algorithms, i.e., back propagation, the quasi-Newton and Levenberg-Marquardt. The values of root mean square (RMS) error and determination coefficient (*R*^2^) were adopted as evaluation criteria for ANNs. The minimum value of the RMS error with the highest value of the determination coefficient was observed for a multi-layer ANN with eight neurons in the hidden layer trained using the quasi-Newton algorithm. The analysis of response surfaces allowed the relationship between the input parameters and the COF to be found. Increasing the load at constant kinematic viscosity of the lubricant reduces the value of the COF. The lowest value of the COF during the friction tests of sheets was provided by oil with a low density and at the same time high kinematic viscosity. High values of COF were visible during friction occurring when using oil with both high-density and, at the same time, high kinematic viscosity. 

## 2. Materials and Methods

### 2.1. Material

Ti-6Al-4V titanium alloy sheets with a thickness of 0.5 mm were used as test material. This two-phase α + β alloy is used primarily in the aviation, space, automotive and medicine industries. Its mechanical properties are shaped by heat and plastic treatment. Table A1 in Appendix A shows the chemical composition of Ti-6Al-4V material. The Ti-6Al-4V alloy is characterized by good plastic and strength properties. It has the same strength as steel, but its density is about 40% lower. It is easily cold worked and is resistant to corrosion at room temperature as well as industrial temperature. The Ti-6Al-4V alloy is used in the production of aircraft engines and airframe support structures.

The roughness parameters of the sheet surface as-received were measured using the Bruker Contour GT 3D optical measuring tool (Billerica, MA, USA) in accordance with EN ISO 25178. The basic parameters of surface roughness include: the average height of the area selected Sa = 0.23 μm, maximum height of the area selected Sz = 2.03 μm, spatial total height St = 2.24 μm, maximum peak height of the area selected Sp = 1.14 μm, maximum valley depth of the area selected Sv = 1.10 μm.

### 2.2. Strip DrawingTest

**Figure 1:**
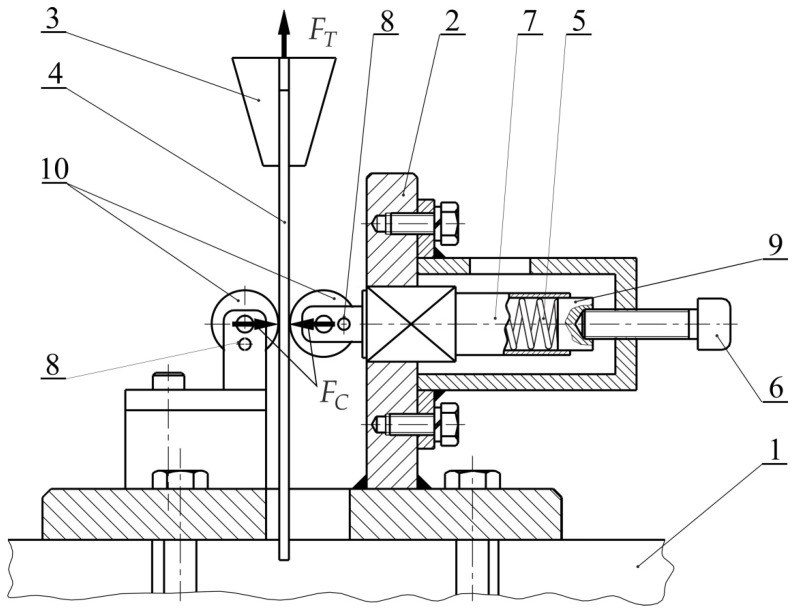
Schematic diagram of friction simulator: 1—bottom grip, 2—base, 3—upper grip, 4—specimen, 5—working rollers, 6—bolt, 7—mandrel, 8—fixing pin, 9—teflon insert, 10—spring.

Figure 1 shows a friction simulator that was mounted on a Zwick/Roell Z100 testing machine (Ulm, Germany). The device frame was mounted in the lower holder of the machine, while flat sheets with a width of *w* = 18 mm were used as the test material. In the test, strips were pulled between fixed cylindrical counter-samples. A friction simulator frame was mounted in the lower bracket of the machine frame, and one of the ends of the sheet metal is mounted in the upper bracket of the machine. The pressure of the rollers was exerted on the sample through a Teflon insert and a working spring. In the test, the spring pressure was achieved by tightening the bolt. Six levels of load between 50 and 200 N were investigated. An average surface roughness of the set of rollers that was used for the test was Ra = 0.32 μm.

The value of the COF was estimated for stabilized ranges of the friction force (excluding the range of the increasing the load force to a specific level (50, 80, 110, 140, 170 and 200 N) according to the following equation

(1) $$\mu =\frac{F_{T}}{2F_{C}}$$

where *F_c_* is the clamping force and *F_t_* is the friction force.

The tests for the specific experiment were repeated three times and the mean COF value was determined. Plan of experiments is listed in Table A2, Table A3, Table A4, Table A5, Table A6, Table A7, Table A8, Table A9 and Table A10. The detailed procedure for the determination of the COF using the friction simulator shown in Figure 1 can be found in a recent paper by the authors [52].

Before the friction tests, the samples and counter-samples were cleaned with acetone. Friction tests were performed under lubricated conditions. In these conditions, five kinds of vegetable oil: palm oil (Ölmühle Solling GmbH, Boffzen, Germany, expiration date: 8 June 2021), olive oil (Sovena Espana SA, Sevilla, Spain, expiration date: 16 September 2021), rapeseed oil (Schalk Mühle GmbH & Co KG, Steiermark, Austria, expiration date 22 April 2021), sunflower oil (SC Argus SA, Constanta, Romania, expiration date: 2 July, 2021) and soybean oil (Basso Fedele e figli s.r.l., Avellino, Italy, expiration date: 23 July 2021) were used. Moreover, and four types of synthetic oil (L-AN 46, L-HL 46, SAE 10W-40, SAE 75W-85) were used. Oils were selected based on the suggestions included in the literature [24,53,54]. The selection also took into account the low cost of these oils and their general availability, and the fact that interest had been shown in their potential for reducing the friction coefficient of titanium sheets [55,56]. Table 1 presents the basic physico-chemical properties of the tested oils.

### 2.3. Regression Model

Quadratic multivariate analysis of variance was used as a tool for determining the relationship between the friction conditions and the value of the COF. The interaction between all factors were fitted with a polynomial that accurately expresses the relationships between all input factors and response values.

When selecting the factors affecting COF, one should take into account the requirements related to the construction of the regression model (RM) describing their impact. These requirements come down to the selection of factors that significantly affect the COF, and at the same time are independent of each other. In the RM analysis the following factors were included: kinematic viscosity, oil density and load (Table 2).

In general, numeric variables have ranges that vary widely. During ANOVA, the differences in the range of individual variables may cause variables with larger ranges to have a greater impact on the COF value. The input data were normalized using the *min-max* function, which transforms the input data values into an interval (*N_min_*, *N_max_*), according to Equation (2):

(2) $$D=\frac{\left(D-min\right)}{max-min}\left(N_{max}-N_{min}\right)+N_{min}$$

where *D*—value of the variable subjected to normalization and (*min*, *max*) is the interval in which the original data are contained.

The explained variable was the value of the COF. The minimum and maximum values of the new interval are designated as Coded Low and Coded High, respectively. The coded values for each input variable are listed in Table 3, Table 4 and Table 5.

Tests for significance have been conducted on individual model coefficients. In backward elimination method, we start with a model in which all independent variables are present. At each step, the independent variable with the highest probability corresponding to Fisher’s parameter *F* is removed from the model if the probability (*p*-value) is sufficiently high (in this research not less than 0.10). In other words, it involves the determination of the *p*-value or probability value, usually relating the risk of falsely rejecting a given hypothesis [58]. The procedure is finished when there are no more variables in the regression equation that meet the removal criteria.

The test for significance of the regression model is performed by calculating the *F*-ratio, which is the ratio between the regression mean square and the mean square error [58]. The *F*-ratio is the ratio of the variance due to the effect of a factor and the variance due to the error term. This ratio is used to measure the significance of the RM under investigation with respect to the variance of all the terms included in the error term at the desired significance level *α* = 0.05.

A popular method of identifying a typical observation in multiple regression analysis is the Cook’s distance method, which compares the degree of fit to the data for two models: the full model, taking into account all observations from the set of observations, and for the model built on the data set in which the one, selected *i*-th observation, is omitted [59]:

(3) $$D_{i}=\frac{\sum_{j=1}^{n}\left({\hat{Y}}_{j}-{\hat{Y}}_{j\left(i\right)}\right)}{m\cdot MSE}$$

where *m* is the number of parameters in the model, *MSE* is the mean square error of the model, ${\hat{Y}}_{j}$ is the predicted value of the *Y* variable, *j* is case number, ${\hat{Y}}_{j\left(i\right)}$ is the predicted value of the *Y* variable in the model built on the set from which the observation number *i* was temporarily excluded.

**Figure 2:**
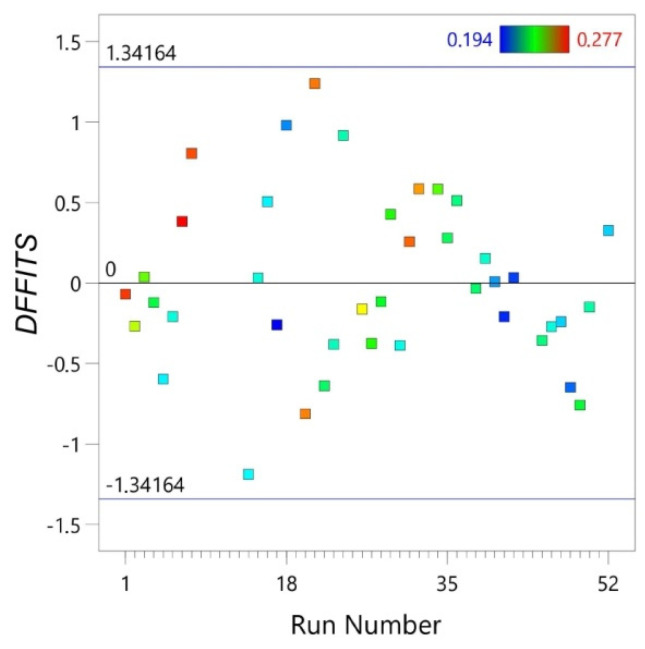
Difference of fits (*DFFITS*) vs. run number.

*DFFITS* is diagnostic meant to show how influential a point is in a statistical regression. Figure 2 shows that all the points in the statistical regression are influential because they are located within the range −1.34164 and +1.34164.

### 2.4. ANN Modeling

One-way multi-layer networks were used for data analysis. The values of nominal pressure, density of lubricants and the viscosity of lubricants were selected as input parameters to the network. On the other hand, the value of COF was expected at the output of the network. The selection of the structure of the neural network depends on the complexity of the problem, in the form of the number of explanatory (input) and explained (output) variables and the size of the training set. Due to the lack of clear guidelines for building a neural network architecture for a specific problem, the article tested the prediction abilities of three neural networks with one hidden layer and a different number of neurons in this layer. Statistica program release 4.0 E Neural Networks (Statsoft Inc., Tulsa, OK, USA) [60] was used to build and analyse ANNs.

The network learning process was carried out using three algorithms, the BP, (qN) and (LM) algorithms. The input data were normalized using the *min-max* function, which transforms the input data values into an interval (*N_min_*, *N_max_*), according to Equation (2).

In the investigations the ANNs were trained based on the results of experimental tests. Overall 15% of the data included in the training set was assigned to the verification set. Data from this set are used to provide independent control of the convergence of the learning process. As a result, of the learning process, the trained neural network acquires the ability to predict the value of the output signal based on the sequence of input signals and the corresponding output signals presented during the learning process. The task of the training algorithm is to select threshold values and weights of neurons in order to minimize the global error of the ANN.

On the basis of the literature analysis [61,62,63], two parameters were adopted as network quality indicators, the root mean square (*RMS*) error (Equation (4)) and the determination coefficient *R*^2^ (Equation (5)):

(4) $$RMS=\sqrt{\frac{1}{n}\overset{n}{\underset{i=1}{\sum }}{\left|a_{j}-p_{j}\right|}^{2}}$$

(5) $$R^{2}=1-\left(\frac{\sum_{i=1}^{n}{\left(a_{j}-p_{j}\right)}^{2}}{\sum_{i=1}^{n}{\left(p_{j}\right)}^{2}}\right)$$

where *a* is the actual value, *p* is the predicted value, and *n* is the number of training sets.

Network training is a key stage in gaining generalization capabilities from a neural network. There are three main training algorithms of multi-layer ANNs: back propagation (BP), the quasi Newton (qN) and the Levenberg-Marquardt (LM).

The back propagation algorithm definitely dominates among the methods of training unidirectional multi-layer networks. The name of the method reflects the principle of its operation, which consists of “transferring” the error committed by the network in the direction from the output layer to the input layer (i.e., backwards to the direction of information flow). 

The Levenberg-Marquardt algorithm is a fast-convergent algorithm. Its computational complexity is not very large and its implementation is simple [64]. The work principle of the LM algorithm is based on the least-squares method [65]. The LM algorithm, also known as the damped least-squares method, works without computing the exact value of the Hessian matrix of the error function. The LM regularization method consists of replacing the Hessian matrix with its approximation based on gradient calculations with a properly selected regularization factor. The algorithm of the LM method approximates the Hessian of the error function by means of an appropriate transformation of the residual matrix and Jacobian. Jacobians are used to determine the sensitivity of the network outputs [66].

In the quasi-Newton method, the Hessian of the minimized error function is approximated by analyzing successive gradient vectors. The variable-metric method assumes that the error function can be approximated by a quadratic function in the neighborhood of the local optimum. The fact that the Hessian satisfies the condition of positive definiteness at each step of the ANN’s training makes the qN method one of the best methods for optimizing multivariable functions. Due to the high computational complexity related to the necessity to calculate *n*^2^ elements of Hessian, this method is recommended for relatively not very complex neural networks.

## 3. Results and Discussion

### 3.1. Strip Drawing Test

**Figure 3:**
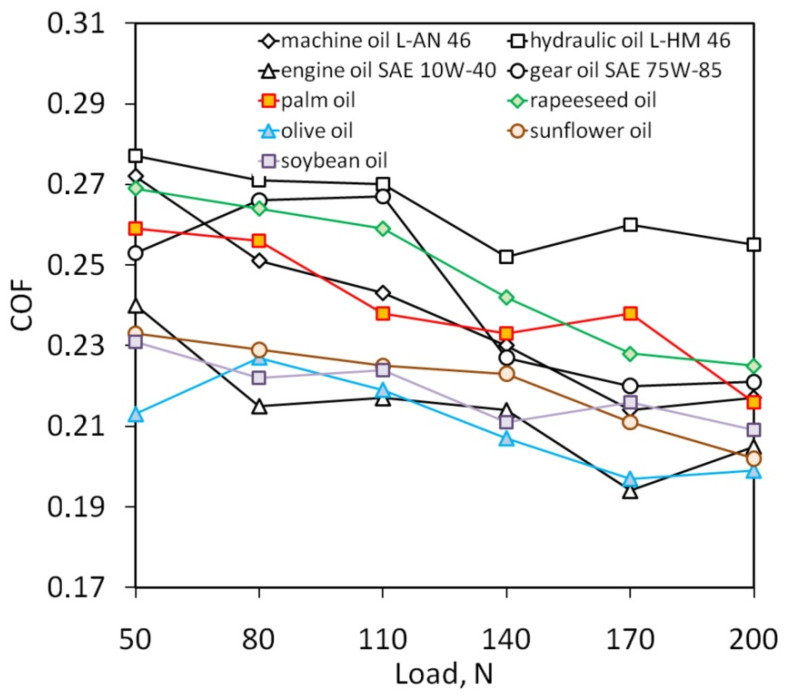
Effect of load on the COF of Ti-6Al-4V titanium alloy sheets.

The results of the strip drawing test are listed in Table A2, Table A3, Table A4, Table A5, Table A6, Table A7, Table A8, Table A9 and Table A10. Increasing the load during the strip drawing test causes a clear tendency to reduce the coefficient of friction (Figure 3). Reducing the value of the COF with increasing load can be explained by the non-linear relationship between clamping and friction forces. Beyond a range of loads analyzed, the relationship between friction force and clamping force is not proportional. In the SMF, the contact area between the sheet metal with relatively low hardness and the harder tool material plays an important role in the tribological phenomena in the contact interface. In the strip drawing test the contact area between the cylindrical countersamples and the strip specimen increases non-linearly with increased load. This conclusion was also drawn by Kirkhorn et al. [67]. Despite the above-mentioned difficulties in the interpretation of the COF, the strip drawing test is a primary method for determination of the COF in SMF [52,68,69,70]. The highest value of COF in terms of the loads considered was observed during lubrication with L-HL 46 hydraulic oil. On the other hand, rapeseed and palm oils showed the worst lubricating properties among the vegetable oils in the entire range of loads considered. Apart from the load of 80 N, olive oil is the lubricant which provided the lowest value of COF during the friction process. Several oils present different local trends of COF changes with load. For example, gear oil SAE 75W-85 only showed an increase in COF with increasing pressure in the pressure range 50–110 N. There was a clear reduction in COF with a further increase in load. It is well known that the friction process under lubricated conditions depends on the load, the volume of the valleys between the load-bearing plastically deformed asperities also known as “oil pockets”, the lubricant density and its viscosity.

It very hard to provide an overall interpretation of the interactional effect of load, kinematic viscosity, and density of lubricants on the value of the COF. For this purpose, two methods, classical analysis of variance and artificial neural networks, were used. Based on the training process, ANNs enable one to analyse multidimensional problems with a large number of independent variables. Neural networks do not require knowledge of the nature of the relationships between the variables. Based on the numerical values entered, they are able to generalize the interactions between the input parameters and the output variable.

### 3.2. ANOVA Analysis

The multivariant quadratic regression was used to determine the polynomial quadratic regression model of the influence of independent variables *A*, *B*, and *C* on the value of COF. 

The data for the value of COF obtained in each run was analyzed using the software Design-Expert® 10.0 and fitted into multiple non-linear regression models proposed by the following equation (in the coded factor) for the value of COF:

(6) $$\mathrm{COF}=0.0219A-0.0958B-0.0179C+0.2277AB+0.1086A^{2}+0.2188$$

The terms BC and AC were eliminated based on the backward elimination regression for a *p*-value greater than 0.1. The elimination of the terms AC and BC improved the coefficient of determination. Therefore, the ANOVA revealed that the products of AC and BC are not statistically significant in the regression equation.

As far as the coded factors are concerned, Equation (6) can be used to make predictions about the response for given levels of each factor. According to Table 3, high levels of the factors in Equation (6) are coded as +1 and low levels are coded as −1. In relation to actual factors, Equation (6) can be used to make predictions about the response for actual levels of each factor.

The results of the ANOVA for the response surface reduced quadratic model are listed in Table 6. The model *F*-value of 22.13 implies the model is significant. There is only a 0.01% chance that an *F*-value this large could occur due to noise. *p*-values less than 0.0500 indicate the model terms are significant. In this case, *A*, *B*, *C*, *AB*, *A*^2^ are significant model terms. Values greater than 0.1000 indicate the model terms are not significant.

Moreover evaluating the significance of the backward elimination regression model, the adequacy of the models was evaluated by applying the lack-of-fit test. The lack-of-fit test measured the adequacy of the different models based on response surface analysis [71]. The lack-of-fit *F*-value of 0.33 implies the lack of fit is not significant relative to pure error. There is a 98.44% chance that a lack of fit *F*-value this large could occur due to noise.

The total *R*-square of the regression model is equal to *R*^2^ = 0.69 (Table 7). The predicted *R*^2^ of 0.6216 is in reasonable agreement with the adjusted *R*^2^ of 0.6660; i.e., the difference is less than 0.2. The adequacy precision parameter measures the signal to noise ratio. A ratio greater than 4 is desirable. An adequacy precision ratio of 17.560 indicates an adequate signal.

**Figure 4:**
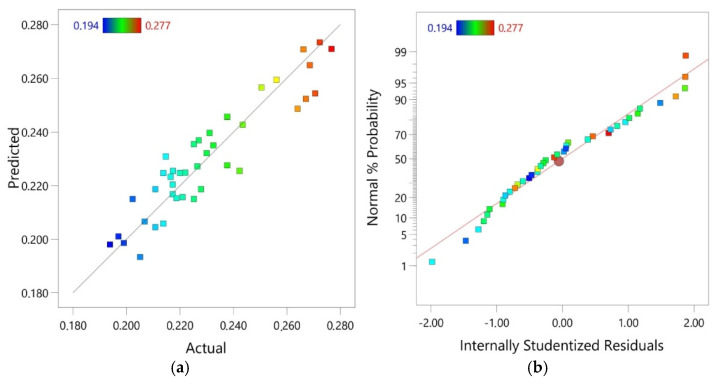
(**a**) linear relationship between predicted and actual values of COF and (**b**) normal probability plot of residuals.

**Figure 5:**
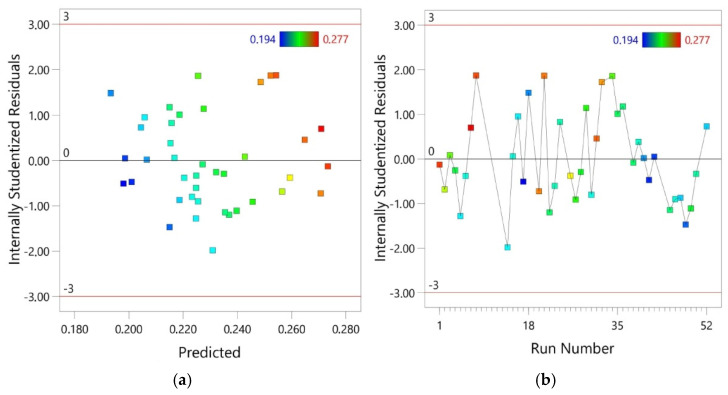
(**a**) relationship between predicted and internally predicted residuals and (**b**) distribution of the internally studentized residuals through the run number.

The RM predicted values were obtained by inserting the values of independent variables into the regression model. The response values were compared with the experimental values. The actual values were relatively close to the predicted straight line regression (Figure 4a). The proportional distribution of data around this line in the entire range of COF changes proves a good correlation between the predicted and actual values. The fact that residuals generally fall on a straight line (Figure 5a,b) implies that the model errors are distributed normally [58]. The results of the diagnostic analysis are supplemented by a normal probability plot of residuals arranged along a straight line (Figure 4b).

**Figure 6:**
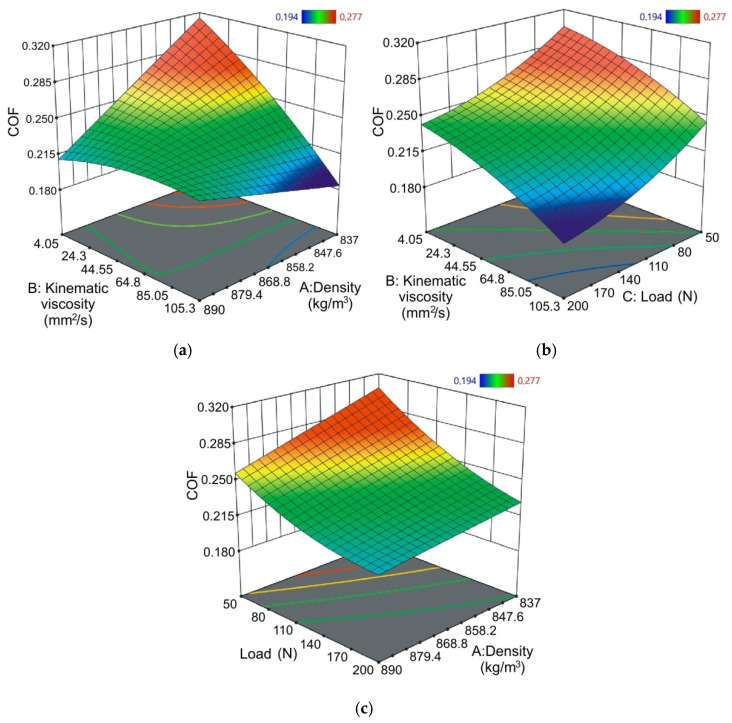
Response surface plots presenting the interaction between (**a**) kinematic viscosity and density of lubricants affecting the COF at constant load, (**b**) kinematic viscosity and load affecting the COF at constant lubricant density and (**c**) load and lubricant density affecting the COF at constant kinematic viscosity of lubricant.

Figure 6 shows the response surfaces and their corresponding contours of the combined effect of kinematic viscosity and density of lubricants (Figure 6a), kinematic viscosity and load (Figure 6b), and lubricant density and load (Figure 6c) on COF. The effect of kinematic viscosity and oil density depends on the effect of the interaction between these parameters (Figure 6a). The lowest value of COF during friction tests of sheets made of Ti-6Al-4V titanium alloy was provided by oil with a low density and at the same time, high kinematic viscosity. The most unfavorable friction conditions occur during friction occurring when using oil with high-density and, at the same time, high kinematic viscosity. Similarly high values of COF are visible for lubrication with oil of low density and at the same time low kinematic viscosity.

Increasing the load at a specific kinematic viscosity of oil reduces the value of COF (Figure 6b). A similar conclusion can be seen for the interaction between load and oil density (Figure 6c). As the force exerted by the load increases, the value of COF decreases. The other problem which makes the interpretation of the friction phenomenon difficult in the SDT is the effect of the real area of the contact surface on the COF in SMF. In metal forming processes the hardness of the workpiece is much less than the hardness of the tool surfaces. Therefore, the mechanism of plowing of asperities plays a dominant role, especially under high pressure conditions. In these conditions, the classical Amontons-Coulomb law is not always satisfied. Therefore, if the relationship between the clamping and friction forces changes, the COF determined from Equation (1) also varies. It is also clear from Figure 6b that a reduction of the kinematic viscosity of the oil leads to an increase in the COF at a specific load.

Viscosity is a property of fluids and plastic solids that characterizes their internal friction resulting from the moving of fluid layers relative to each other. If the viscosity is too low, the lubricant does not provide a sufficient “cushion” between the sliding surfaces. This can lead to problems such as increased friction and wear as well as increased heat generation and oxidation of the material. Too high a viscosity value can lead to insufficient oil flow in their inner layers and an increase in frictional resistance, which in turn increases the temperature in the region of surface asperities.

Sheets made of Ti-6Al-4V alloy are highly susceptible to galling. By increasing the load of the sample, the adhesion mechanism becomes less important, while galling and plowing mechanisms taking on a dominant role. At high pressures, the lubricant is not able to lower the COF of metallic sheets effectively, which are therefore susceptible to galling. However, the lowest values of COF were observed at the highest pressures and at the same time with high kinematic viscosity of the lubricant. Under these conditions, the lubricant was able to produce a mixed lubrication regime in which the friction surfaces are largely separated by a layer of lubricant [72]. A prerequisite for the formation of a mixed lubrication regime on a surface, according to the Stribeck curve, is the presence on the surface of (i) oil pockets which form a large volume, for example, by a roughening mechanism, (ii) lubrication with a high-viscosity lubricant and (iii) high pressures.

**Figure 7:**
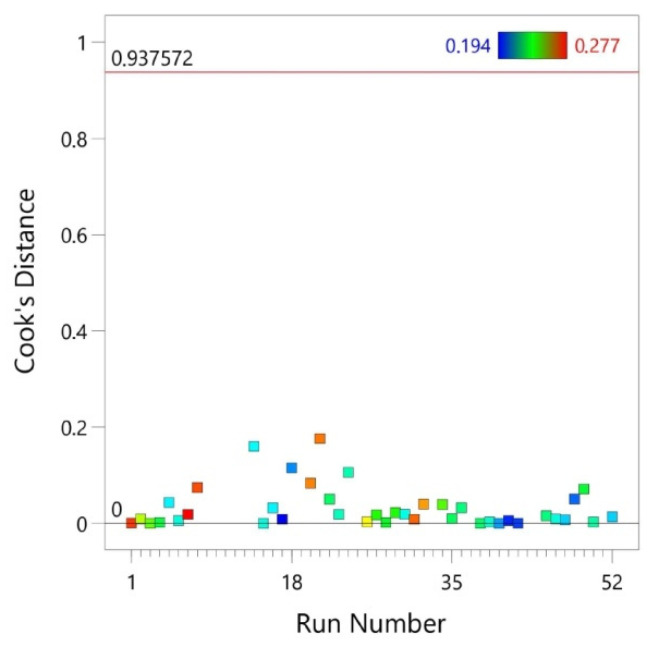
Cook’s distance vs. run number.

Cook’s distance is a measure of the effect of a given case on the regression equation. It shows the difference between the values of the coefficients determined in the regression equation and the calculated values when a given case was excluded from the calculations. In the correct model, all distances should be of the same order, which is confirmed by the results in Figure 7. This means that the given case/cases had no significant influence on the bias of the coefficients of the regression equation.

The *DFFITS* indicates the effect that deleting each observation has on the predicted values of the regression model (Figure 2). The *DFFITS* is a studentized *DFFIT* which is the change in the predicted value for a point, obtained when that point is left out of the regression:

(7) $$DFFITS=\frac{y_{i}-y_{i\left(i\right)}}{S_{\left(i\right)}\sqrt{h_{ii}}}$$

where *h_ii_* is the leverage for the point, *s*_(*i*)_ is the standard error estimated without the point in question, $\hat{y_{i}}$ is the prediction for the point with the point included in the regression and $y_{i\left(i\right)}$ is the prediction for the point without the point included in the regression.

### 3.3. ANN Modeling

**Figure 8:**
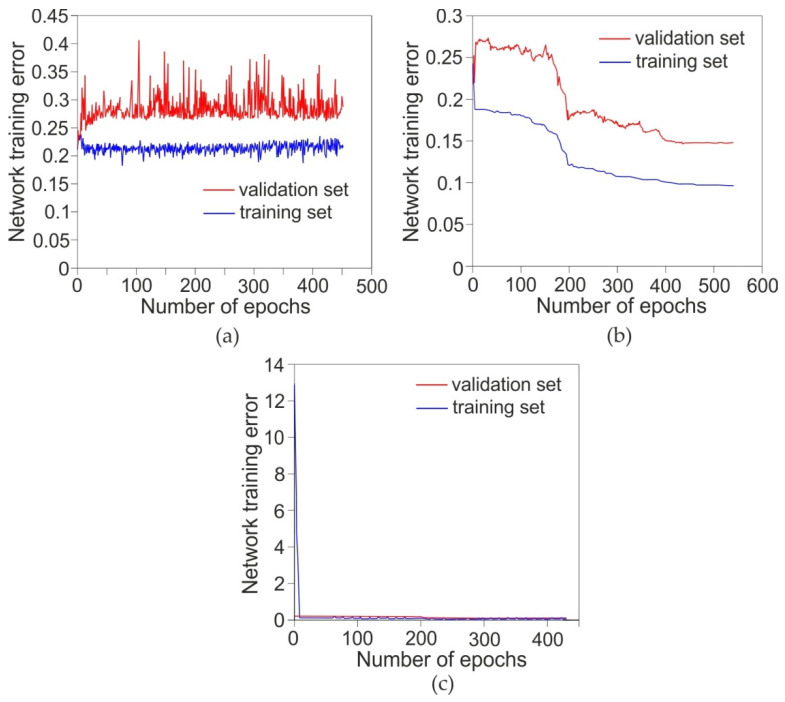
Network training errors for the training set and the validation set during network training with the BP (**a**), qN (**b**) and LM (**c**) algorithms.

Using the Statistica program release 4.0 E Neural Networks (Statsoft Inc., Tulsa, OK, USA), many experiments were carried out with different network architectures. On the basis of the correlation coefficient and the value of the RMS error, the network 3:3-8-1:1 network was selected for further considerations. A network witch such this structure provided the highest value of the correlation coefficient with the lowest RMS error value. The network training process was carried out independently with three algorithms: BP, LM and qN. Training was carried out until no further reduction of the network error observed for the training set was achieved. The training algorithm was stopped at the network error of 0.213, 0.096 and 0.119 for the BP, qN and LM algorithms, respectively. The training process with the BP algorithm was characterised by a saw-shaped course (Figure 8a). This is a typical behavior of error changes when training the network with the BP algorithm, especially in the case of undetermined relations between the input variables and the explained variable. The value of the correlation coefficient for the training set after teaching the network with the BP algorithm was *R*^2^ = 0.5979 (Table 8). Under these conditions, the network RMS error value under these conditions was 0.249 and 0.305 for the training and validation sets, respectively. It is worth noting that the spread of error values for the validation set was much larger (red line in Figure 8a) than for the training set (blue line in Figure 8a). The explanation is that the number amount of data contained in the training set is much larger than in the validation set.

The training process with the quasi-Newton algorithm (Figure 8b) exhibited a different character of change in the network error value. After several epochs similar to the BP algorithm, the qN algorithm provided more than double the reduction in the RMS error value for the training set (RMS = 0.0982) and the validation set (RMS = 0.1493). The LM algorithm (Figure 8c) had the most stable learning process. In a similar manner to learning with the BP algorithm, after about 10 epochs, the algorithm reached a stable minimum error, which in a later stage was only slightly reduced. Moreover, the number of epochs after which no further error reduction occurred is similar to the BP algorithm (Figure 8a). The values of the RMS error for the training and validation sets of the network learned with the LM algorithm were 0.114 and 0.1707 respectively. Therefore a network learned with the qN algorithm was selected for further analysis, which provided the lowest RMS error value for the training set and the highest value of the correlation coefficient (*R*^2^ = 0.91). Apart from the correlation coefficient *R*^2^, an important parameter proving the quality of the ANN is the S.D. ratio parameter (Table 8). It is the quotient of the standard deviation of errors (Error S.D.) and the standard deviation of the value of the explained variable (Data S.D.). This parameter is never negative. However the lower is the value, the better is the quality of the model.

**Figure 9:**
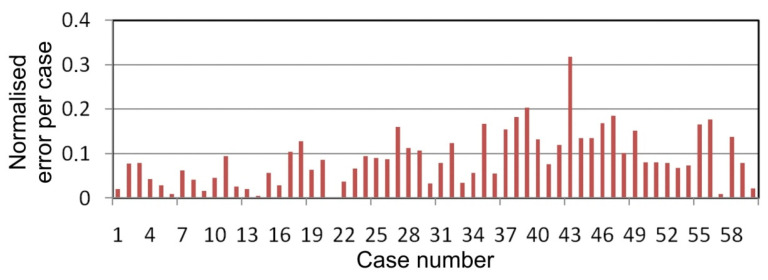
Normalized error per case for 3:3-8-1:1 ANN.

As can be seen from Figure 9 the normalized errors for cases 35–50 are much larger than for cases 1–15. It may be that an improvement in the ANN results could be obtained by carrying out additional friction tests with oils with a kinematic viscosity between the kinematic viscosities of the oils tested in this study. Nevertheless, the results confirmed the potential of ANNs to model the value of the coefficient of friction of Ti-6Al-4V titanium alloy sheets.

## 4. Conclusions

The article presents the results of the application of an ANN to the modeling of the value of the COF of Ti-6Al-4V titanium alloy sheets in a strip drawing test. The following conclusions are drawn from the experimental investigations and ANN modeling:

- As a result of the non-linear relationship between the clamping and the friction force, the value of the COF decreased with increasing pressure force. The relationship between friction force and clamping force is not proportional beyond the range of loads analyzed. Moreover, the contact area between the cylindrical roller and the flat specimen increases non-linearly with increased load. It is well known that the contact area plays a key role in the friction process in plastic working, contrary to friction in the kinematic pairs.
- The highest value of the COF in terms of the considered loads was observed during lubrication with L-HL 46 hydraulic oil.
- Apart from the load of 80 N, olive oil is the lubricant which provided the lowest value of COF during the friction process.
- Increasing the load at a constant value of kinematic viscosity of lubricant reduces the COF value.
- The lowest value of COF during friction tests of sheets made of Ti-6Al-4V titanium alloy was provided by oil with a low density and at the same time high kinematic viscosity.
- The minimum value of RMS error with the highest value of correlation coefficient was observed for a multi-layer network with eight neurons in a hidden layer learned with the qN algorithm.
- With all the algorithms investigated during the training process, a higher value of the network error was noted with the validation set than with the training set since the latter contains 85% of all experimental data sets.

## Figures and Tables

**Table 1:** Basic physico-chemical properties of tested oils [57].

Oil Type	Density *ρ*, kg/m^3^	Kinematic Viscosity *η_k_*, mm^2^/s	Flash Point *T_f_*, °C
olive oil	890	4.52	179
sunflower oil	883	4.45	185
palm oil	875	5.72	165
soybean oil	891	4.05	176
rapeseed oil	880	4.45	62
engine oil SAE 10W-40	872	105.3	220
machine oil L-AN 46	875	43.9	232
gear oil SAE 75W-85	837	64.6	202
hydraulic oil L-HL 46	877	44.2	227

**Table 2:** Factors and levels for ANOVA analysis.

Factor	Name	Unit	Type	Minimum	Maximum	Coded Low	Coded High	Mean	Standard Deviation
*A*	Density	kg/m^3^	Numeric	837	891	−1 ↔ 837	+1 ↔ 891	875.22	15.00
*B*	Kinematic viscosity	mm^2^/s	Numeric	4.05	105.3	−1 ↔ 4.05	+1 ↔ 105.3	31.24	34.42
*C*	Load	N	Numeric	50	200	−1 ↔ 50	+1 ↔ 200	125	51.72

**Table 3:** Coded values of the densities of the lubricants.

Real value	837	872	875	877	880	883	890	891
Coded value	−1	0.296	0.407	0.481	0.592	0.703	0.963	1

**Table 4:** Coded values of the kinematic viscosity of the lubricants.

Real value	4.05	4.45	4.52	5.72	44.2	43.9	64.6	105.3
Coded value	−1	−0.992	−0.990	−0.967	−0.207	−0.212	0.196	1

**Table 5:** Coded values of the load.

Real value	50	80	110	140	170	200
Coded value	−1	−0.6	−0.2	0.2	0.6	1

**Table 6:** Results of ANOVA for the response surface reduced quadratic model.

Source	Sum of Squares	Degrees of Freedom	Mean Square	F-Value	*p*-Value	Meaning
Model	0.0185	5	0.0037	22.13	<0.0001	significant
*A*—Density	0.0012	1	0.0012	6.90	0.0115	-
*B*—Kinematic viscosity	0.0064	1	0.0064	38.48	<0.0001	-
*C*—Load	0.0081	1	0.0081	48.53	<0.0001	-
AB	0.0052	1	0.0052	30.83	<0.0001	-
A^2^	0.0034	1	0.0034	20.55	<0.0001	-
Residual	0.0080	48	0.0002	-	-	-
Lack of Fit	0.0056	42	0.0001	0.3329	0.9844	not significant
Pure Error	0.0024	6	0.0004	-	-	-
Correlation Total	0.0265	53	-	-	-	-

**Table 7:** Fit statistics of the regression model.

Standard Deviation	0.0129	*R* ^2^	0.6975
Mean	0.2332	Adjusted *R*^2^	0.6660
Coefficient of Variation %	5.54	Predicted *R*^2^	0.6216
		Adequacy Precision	17.5598

**Table 8:** Basic regression statistics of the ANNs analyzed and trained using the BP (**a**), qN (**b**) and LM (**c**) algorithms.

**BP (a)**		
**Parameter**	**Training set**	**Validation set**
Data mean	0.3828	0.3783
Data S. D.	0.2395	0.2252
Error mean	−0.1615	−0.1333
Error S. D.	0.1921	0.2870
Abs E. mean	0.1931	0.2582
S. D. ratio	0.8019	1.2744
Correlation	0.5979	0.0768
**qN (b)**		
**Parameter**	**Training set**	**Validation set**
Data mean	0.3828	0.3783
Data S. D.	0.2395	0.2252
Error mean	−0.0004	0.0146
Error S. D.	0.0993	0.1551
Abs E. mean	0.0819	0.1276
S. D. ratio	0.4145	0.6891
Correlation	0.9100	0.7247
**LM (c)**		
**Parameter**	**Training set**	**Validation set**
Data mean	0.3828	0.3783
Data S. D.	0.2395	0.2252
Error mean	0.00005	0.0473
Error S. D.	0.1157	0.1717
Abs E. mean	0.1009	0.1503
S. D. ratio	0.4830	0.7624
Correlation	0.8756	0.6514

## Data Availability

The data presented in this study are available on request from the corresponding author.

## Appendix A

**Table A1:** Chemical composition of the Ti-6Al-4V titanium alloy examined (in wt.%) [73].

Al	V	O	Fe	H	C	N	Ti
5.5	3.5	<0.2	<0.3	<0.0015	<0.08	<0.5	remainder

**Table A2:** Results of the strip drawing test conducted with lubrication using L-AN 46 machine oil.

Number of Experiment	Force, N	COF	COF (Average Value)
1	50	0.268	0.272
2	50	0.265	
3	50	0.283	
4	80	0.250	0.251
5	80	0.242	
6	80	0.261	
7	110	0.245	0.243
8	110	0.235	
9	110	0.25	
10	140	0.229	0.230
11	140	0.224	
12	140	0.237	
13	170	0.200	0.214
14	170	0.219	
15	170	0.223	
16	200	0.217	0.217
17	200	0.21	
18	200	0.224	

**Table A3:** Results of the strip drawing test conducted with lubrication using L-HL 46 hydraulic oil.

Number of Experiment	Force, N	COF	COF (Average Value)
19	50	0.282	0.277
20	50	0.268	
21	50	0.281	
22	80	0.273	0.271
23	80	0.263	
24	80	0.275	
25	110	0.278	0.270
26	110	0.259	
27	110	0.273	
28	140	0.246	0.252
29	140	0.245	
30	140	0.259	
31	170	0.261	0.260
32	170	0.248	
33	170	0.271	
34	200	0.255	0.255
35	200	0.25	
36	200	0.259	

**Table A4:** Results of the strip drawing test conducted with lubrication using SAE 10W-40 engine oil.

Number of Experiment	Force, N	COF	COF (Average Value)
37	50	0.247	0.240
38	50	0.228	
39	50	0.245	
40	80	0.208	0.215
41	80	0.213	
42	80	0.223	
43	110	0.219	0.217
44	110	0.211	
45	110	0.221	
46	140	0.217	0.214
47	140	0.204	
48	140	0.221	
49	170	0.196	0.194
50	170	0.183	
51	170	0.203	
52	200	0.210	0.205
53	200	0.198	
54	200	0.207	

**Table A5:** Results of the strip drawing test conducted with lubrication using SAE 75W-85 engine oil.

Number of Experiment	Force, N	COF	COF (Average Value)
55	50	0.258	0.253
56	50	0.244	
57	50	0.257	
58	80	0.269	0.266
59	80	0.259	
60	80	0.270	
61	110	0.272	0.267
62	110	0.257	
63	110	0.272	
64	140	0.183	0.227
65	140	0.224	
66	140	0.274	
67	170	0215	0.220
68	170	0.219	
69	170	0.226	
70	200	0.216	0.221
71	200	0.218	
72	200	0.229	

**Table A6:** Results of the strip drawing test conducted with lubrication using palm oil.

Number of Experiment	Force, N	COF	COF (Average Value)
73	50	0.260	0.259
74	50	0.253	
75	50	0.264	
76	80	0.253	0.256
77	80	0.251	
78	80	0.264	
79	110	0.239	0.238
80	110	0.229	
81	110	0.246	
82	140	0.234	0.233
83	140	0.227	
84	140	0.238	
85	170	0.239	0.238
86	170	0.231	
87	170	0.243	
88	200	0.218	0.216
89	200	0.209	
90	200	0.221	

**Table A7:** Results of the strip drawing test conducted with lubrication using rapeseed oil.

Number of Experiment	Force, N	COF	COF (Average Value)
91	50	0.269	0.269
92	50	0.265	
93	50	0.273	
94	80	0.267	0.264
95	80	0.268	
96	80	0.257	
97	110	0.260	0.259
98	110	0.252	
99	110	0.265	
100	140	0.241	0.242
101	140	0.237	
102	140	0.248	
103	170	0.227	0.228
104	170	0.224	
105	170	0.233	
106	200	0.229	0.225
107	200	0.215	
108	200	0.231	

**Table A8:** Results of the strip drawing test conducted with lubrication using olive oil.

Number of Experiment	Force, N	COF	COF (Average Value)
109	50	0.214	0.213
110	50	0.206	
112	50	0.219	
112	80	0.226	0.227
113	80	0.221	
114	80	0.234	
115	110	0.217	0.219
116	110	0.212	
117	110	0.227	
118	140	0.213	0.207
119	140	0.192	
120	140	0.216	
121	170	0.195	0.197
122	170	0.191	
123	170	0.205	
124	200	0.202	0.199
125	200	0.193	
126	200	0.203	

**Table A9:** Results of the strip drawing test conducted with lubrication using sunflower oil.

Number of Experiment	Force, N	COF	COF (Average Value)
127	50	0.277	0.233
128	50	0.215	
129	50	0.207	
130	80	0.226	0.229
131	80	0.223	
132	80	0.238	
133	110	0.231	0.225
134	110	0.213	
135	110	0.231	
136	140	0.223	0.217
137	140	0.204	
138	140	0.224	
139	170	0.215	0.211
140	170	0.202	
141	170	0.216	
142	200	0.207	0.202
143	200	0.194	
144	200	0.205	

**Table A10:** Results of the strip drawing test conducted with lubrication using soybean oil.

Number of Experiment	Force, N	COF	COF (Average Value)
145	50	0.229	0.231
146	50	0.225	
147	50	0.239	
148	80	0.220	0.222
149	80	0.216	
150	80	0.23	
151	110	0.229	0.224
152	110	0.227	
153	110	0.216	
154	140	0.212	0.211
155	140	0.203	
156	140	0.217	
157	170	0.208	0.216
158	170	0.221	
159	170	0.219	
160	200	0.213	0.209
161	200	0.198	
162	200	0.216	
